# Brain functional connectivity is altered in patients with Takotsubo Syndrome

**DOI:** 10.1038/s41598-019-40695-3

**Published:** 2019-03-12

**Authors:** Ana Rita Silva, Ricardo Magalhães, Carina Arantes, Pedro Silva Moreira, Mariana Rodrigues, Paulo Marques, Jorge Marques, Nuno Sousa, Vitor Hugo Pereira

**Affiliations:** 1 Life and Health Sciences Research Institute (ICVS), School of Medicine, University of Minho, Braga, Portugal; 2 ICVS/3B’s - PT Government Associate Laboratory, Braga, Portugal; 3 Clinical Academic Center (2CA – Braga), Braga, Portugal; 4 Cardiology Department, Hospital of Braga, Braga, Portugal; 5 Algoritmi Centre, University of Minho, Braga, Portugal

## Abstract

Takotsubo syndrome (TTS) is an acute, reversible cardiomyopathy. The central autonomic nervous system (ANS) is believed to play a role in this disease. The aim of the present study was to investigate the patterns of brain functional connectivity in a sample of patients who had experienced a previous episode of TTS. Brain functional connectivity, both at rest and in response to the stressful stimulus of topical cold stimulation, was explored using functional magnetic resonance imaging (fMRI), network-based statistics (NBS) and graph theory analysis (GTA) in a population consisting of eight patients with a previous episode of TTS and eight sex- and age-matched controls. At rest, a network characterized by increased connectivity in the TTS group compared to controls and comprising elements of the central ANS was identified. GTA revealed increased local efficiency, clustering and strength in regions of the bilateral hippocampus in subjects with a previous episode of TTS. When stressed by local exposure to cold, the TTS group differed significantly from both a pre-stress baseline interval and from the control group, showing increased connectivity in a network that included the left amygdala and the right insula. Based on the results, patients with TTS display a reorganization of cortical and subcortical networks, including areas associated with the emotional response and autonomic regulation. The findings tend to support the hypothesis that a deregulation of autonomic control at the central level plays a significant role in this syndrome.

## Introduction

Takotsubo syndrome (TTS) cases have increasingly been reported, but its pathophysiology remains uncertain. The most compelling explanation for TTS is that it occurs as a consequence of catecholamine-induced myocardial stunning^1–3^. This hypothesis is supported by studies showing elevated blood levels of these hormones in patients with TTS^1,4^ as well as by the observation that exogenously administered catecholamines induce cardiac abnormalities similar to those observed in patients with TTS^5–7^. Patients with TTS have recently been reported to exhibit significant increases in sympathetic nerve activity and decreased parasympathetic modulation in both acute^3^ and chronic phases^8^ of the disease, thus highlighting the etiological importance of the catecholaminergic system and its primary regulator – the autonomic nervous system (ANS) – in this syndrome.

In the present study, we sought to determine whether the central ANS network in patients who previously experienced an episode of TTS is functionally different from the network in controls. Previous reports have described the occurrence of TTS following insular stroke, further suggesting that the central ANS may play a role in the pathophysiology of this syndrome^9–11^. In addition, our group has previously shown (using functional magnetic resonance imaging (fMRI) in a small sample of four patients) that the response of the insular cortex to an autonomic challenge (Valsalva maneuver) was altered in patients with TTS compared to healthy age-matched individuals^12^. Other researchers have observed increased connectivity in the precuneus region and decreased connectivity in the ventromedial prefrontal cortex of patients with TTS compared to healthy controls during the resting state^13^.

Klein *et al*. identified alterations in anatomical and neurophysiological measures in brain regions involved in the emotional-autonomic control system as predictors of TTS^14^. The same group more recently revealed anatomical differences between patients with TTS and healthy control subjects in elements of the limbic network comprising the insula, amygdala, cingulate cortex, and hippocampus^15^.

However, none of these studies analyzed whether functional connectivity is altered in patients with TTS in response to a stressful stimulus. Our study aims to provide further insights into the brain connectome of patients who previously experienced an episode of TTS, both in the resting state and during cold exposure (an activator of the autonomic system), to identify potential functional signatures of TTS and address the aforementioned issue. A structural analysis was also performed to identify anatomical correlates of functional differences between patients with TTS and controls.

## Results

### Characterization of the study population

The study sample was selected from a database of twenty-nine patients with an established diagnosis of TTS from the Hospital of Braga. The eight patients selected for this study (inclusion criteria are described in the Methods section) did not differ significantly from the original cohort with respect to age (*t*(35) = 1.77, *p* = 0.086), years of education (*U* = 88.0, *p* = 0.507), Hospital Anxiety and Depression Scale (HADS) score (*t*(35) = 0.257, *p* = 0.799), 10-item Perceived Stress Scale (PSS-10) score (*t*(35) = 0.692, *p* = 0.493) or clinical parameters (Table 1).

**Table 1:** Demographic profile and clinical features of patients with TTS (*n* = 8) at admission.

Category	*N*	Column N	mean	SD
Age			55.3	7.09
Emotional trigger	5	62.5%		
Chest pain	7	87.5%		
Dyspnea	4	50.0%		
Heart rate (bpm)			90.5	22.8
Systolic blood pressure (mmHg)			112.9	16.6
Diastolic blood pressure (mmHg)			76.8	11.9
ST-elevation on electrocardiogram	5	62.5%		
Apical ballooning on left ventriculography	7	87.5%		
Ejection fraction (%)			35.5	5.5
Troponin (peak - ng/mL)			2.64	1.098

*n* = sample size; SD = standard deviation.

Compared with controls, however, patients with TTS reported a significantly higher level of depressive symptoms [HADS subscore (*t*(14) = 2.24, *p* = 0.042) and HADS total score (*t*(14) = 2.27, *p* = 0.039)]. A trend toward increased anxiety symptoms [HADS subscore (*t*(14) = 1.12, *p* = 0.281)] and perceived stress [PPS-10 (*t*(8.4) = 1.84, *p* = 0.102)] was observed in patients with TTS. Neither of these changes achieved statistical significance (Table 2).

**Table 2:** Sociodemographic profile and assessments of anxiety, depression and perceived stress in the TTS and control groups.

Category	TTS (n = 8)		Control (n = 8)		Statistics		
	mean	SD	mean	SD			
Age (years)	58.6	7.44	58.6	7.44			
Education (years)	6.5*	[4–11]*	11.5	2.51	*U* = 14	*p* = 0.053	*r* = −0.48
**HADS**							
Anxiety subscore	10.3	6.23	6.00	2.00	*t*(8.4) = 1.84	*p* = 0.102	*d* = 0.93
Depression subscore	7.50	3.59	3.88	2.85	*t*(14) = 2.24	*p* = 0.042	*d* = 1.12
Total score	17.8	8.80	9.88	4.32	*t*(14) = 2.27	*p* = 0.039	*d* = 1.14
PSS-10 score	16.8	8.41	12.6	6.11	*t*(14) = 1.12	*p* = 0.281	*d* = 0.57

*Data without a normal distribution are presented as medians and [interquartile range]. *n* = sample size; SD = standard deviation; *p* = p-value; *t* = Student’s t-test; *U* = Mann-Whitney U-test*; d* = *Cohen’s d; r* = effect size.

### Resting state analysis

To study the brain functional connectivity of subjects at rest, a connectomics approach using network-based statistics (NBS) was employed. This analysis corrects for the familywise error (FWE) for mass univariate testing. It was complemented by graph theory analysis (GTA) to identify putative nodes that play important roles in TTS.

**Figure 1:**
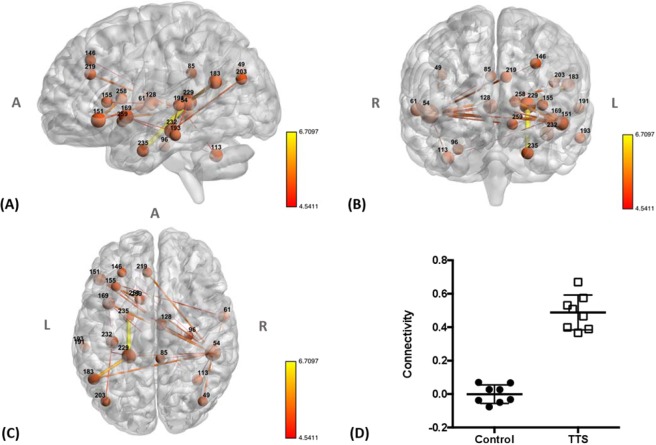
(**A**) Sagittal, (**B**) coronal and (**C**) axial views of the network identified using NBS (edge *p/t*-threshold = 0.001/4.14, network *p* = 0.041 and edge *p/t*-threshold = 0.0005/4.5, network *p* = 0.014) show increased connectivity in patients with TTS compared to controls during rs-fMRI acquisition. Lines (edges) represent functional connections. Brown spheres represent the centroid of each node and are scaled according to the sum of t-statistic values over all of its significant connections. Hotter colors for connections indicate increasing statistical significance. A – anterior, R – right, L – left. (For further details, see Supplemental Table 1). (**D**) Plot showing functional connectivity (the average z-transformed correlation coefficient for the significant network) in patients with TTS and controls during rs-fMRI. Error bars indicate standard errors.

By applying NBS, we identified a resting state network consisting of 21 nodes (#49, #54, #61, #85, #96, #113, #128, #146, #151, #155, #169, #183, #191, #193, #203, #219, #229, #232, #235, #258, and #259) for the following thresholds: edge *p/t*-threshold = 0.001/4.14, network *p* = 0.041 and edge *p/t*-threshold = 0.0005/4.5, network *p* = 0.014. In this network, patients with TTS displayed increased functional connectivity compared to controls (Fig. 1, Table 3, and Supplemental Material 1). The most relevant nodes of this resting state network (the nodes with the highest t-statistic sum for all the connections surviving the threshold), were located in the bilateral superior temporal cortex (#54 and #183), left inferior frontal cortex (#151), left anterior insula (#169), left anterior cingulate cortex (aCC) (#219), left hippocampus (#229 and #232) and left parahippocampal cortex (#235). Within the central ANS, increased functional connectivity was observed between the left anterior insula (#169) and the left hippocampus (#232) as well as the right superior temporal cortex (#54). Enhanced connectivity was also identified between the left aCC (#219) and right superior temporal cortex (#54). The right superior temporal cortex (#54) exhibited the greatest number of enhanced connections in the TTS group compared to controls. No network was identified in which patients with TTS showed reduced functional connectivity compared to controls. In addition, no significant associations between the mean functional connectivity values and HADS (*p* = 0.33) or PSS (*p* = 0.73) scores were found.

**Table 3:** NBS results for the resting state analysis.

*Edge p/t*-threshold (df = 14)	Network *p*	*n* edges	*n* nodes	*g*
0.005/3.32	0.062	240	146	5.92
0.001/4.14	0.041	39	36	5.41
0.0005/4.5	0.014	23	21	5.53

df = degrees of freedom, *n* edges = number of edges in the network, *n* nodes = number of nodes in the network, g = Hedge’s g.

A complete description of the results of between-group comparisons of the graph metrics (local clustering, local efficiency and global strength) is presented in Table 4. For simplicity, the results of the Bayesian analysis for those regions of interest (ROIs) in which at least moderate evidence for the alternative model over the null-hypothesis model was obtained are detailed below. This approach has the advantage of enabling researchers to quantify the likelihood of the null hypothesis against the alternative hypothesis, given a prior probability. This method contrasts the typical correction for multiple comparisons from a frequentist perspective, which is likely to introduce inferential arbitrariness. The analyses of the Bayes factors for local clustering revealed moderate evidence for the alternative hypothesis for ROIs #94 (right hippocampus) (*BF*_10_ = 5.54; posterior median of −1.14; 95% confidence interval (CI): [−2.35, −0.09]), #233 (left hippocampus and parahippocampal cortex; *BF*_10_ = 3.95; posterior median of −1.06; 95% CI: [−2.21, −0.07]) and #259 (left caudate nucleus and left putamen; *BF*_10_ = 48.81; posterior median of −1.11; 95% CI: [−2.27, −0.11]). In addition, strong evidence for the alternative hypothesis was obtained for ROI #230 (left hippocampus; *BF*_10_ = 10.48; posterior median of −1.36; 95% CI: [−2.58, −0.30]). Concerning the local efficiency, moderate evidence for the alternative hypothesis model was obtained for ROIs #94 (right hippocampus; *BF*_10_ = 5.80; posterior median of −1.15; 95% CI: [−2.35, −0.14]), #229 (left hippocampus; *BF*_10_ = 3.47; posterior median of −0.97; 95% CI: [−2.10, −0.02]) and #259 (left hippocampus; *BF*_10_ = 3.73; posterior median of −1.00; 95% CI: [−2.17, −0.05]). Very strong evidence for the alternative hypothesis over the null hypothesis was observed for ROI #230 (left hippocampus; *BF*_10_ = 54.48; posterior median of −1.93; 95% CI: [−3.27, −0.58]). An analysis of global strength yielded moderate evidence for the alternative model for ROIs #93 (right hippocampus; *BF*_10_ = 4.01; posterior median of −1.04; 95% CI: [−2.19, −0.01]), #99 (right hippocampus; *BF*_10_ = 5.24; posterior median of −1.15; 95% CI: [−2.33, −0.08]) and #232 (left hippocampus; *BF*_10_ = 5.25; posterior median of −1.13; 95% CI: [−2.31, −0.16]), and very strong evidence for ROI #230 (left hippocampus; *BF*_10_ = 31.90; posterior median of −1.72; 95% CI: [−3.06, −0.59]).

**Table 4:** Results of the graph theory analysis of resting state data.

Node	Brain Region	Clustering			Efficiency			Degree		
		*t*-statistic^a^	*d* ^b^	BF_10_	t-statistic^a^	d^b^	BF_10_	t-statistic^a^	d^b^	BF_10_
5	Frontal_Sup_Medial_R	*t*(14) = −0.76, *p* = 0.462	*d* = −0.378	0.519	*t*(11.11) = −0.933, *p* = 0.371	*d* = −0.467	0.572	*U* = 42, *p* = 0.328	*r* = 0.328	0.433
10	Frontal_Mid_Orb_R	*t*(14) = −1.4, *p* = 0.183	*d* = −0.701	0.81	*t*(14) = −1.43, p = 0.174	*d* = −0.715	0.832	*U* = 34, *p* = 0.878	*r* = 0.878	0.436
15	Cingulum_Ant_R	*t*(14) = −0.46, *p* = 0.655	*d* = −0.228	0.459	*t*(14) = −0.84, p = 0.416	*d* = −0.419	0.541	*t*(14) = −0.66, *p* = 0.52	*d* = −0.33	0.496
20	Insula_R	*t*(14) = −2.1, *p* = 0.055	*d* = −1.047	1.654	*t*(14) = −1.85, p = 0.086	*d* = −0.924	1.253	*U* = 24, *p* = 0.442	*r* = 0.442	0.483
29	Frontal_Sup_R	*t*(14) = −1.8, *p* = 0.094	*d* = −0.899	1.188	*t*(14) = −1.86, p = 0.085	*d* = −0.927	1.262	*U* = 25, *p* = 0.505	*r* = 0.505	0.455
34	Insula_R	*t*(14) = 0.47, *p* = 0.645	*d* = 0.236	0.461	*t*(14) = 0.67, *p* = 0.516	*d* = 0.334	0.497	*t*(14) = 0.26, *p* = 0.8	*d* = 0.129	0.438
35	Insula_R	*t*(14) = −0.32, *p* = 0.757	*d* = −0.158	0.442	*U* = 32, *p* = 1	*r* = 0	0.428	*U* = 38, *p* = 0.574	*r* = 0.574	0.469
36	Insula_R	*t*(14) = −1.44, *p* = 0.171	*d* = −0.721	0.84	*t*(14) = −1.53, *p* = 0.15	*d* = −0.762	0.905	*t*(14) = −0.84, *p* = 0.417	*d* = −0.418	0.541
37	Insula_R	*t*(14) = −1.58, *p* = 0.136	*d* = −0.79	0.955	*t*(14) = −1.74, *p* = 0.104	*d* = −0.87	1.119	*t*(14) = −1.9, *p* = 0.078	*d* = −0.95	1.327
40	Insula_R	*t*(14) = −0.18, p = 0.862	*d* = −0.089	0.432	*t*(14) = 0.08, *p* = 0.934	*d* = 0.042	0.429	*U* = 30, *p* = 0.878	*r* = 0.878	0.452
83	Cingulum_Ant_R	*t*(14) = −0.65, *p* = 0.525	*d* = −0.326	0.494	*t*(14) = −0.65, *p* = 0.528	*d* = −0.323	0.493	*t*(14) = 0.58, *p* = 0.57	*d* = 0.291	0.48
92	ParaHippocampal_R	*t*(14) = −1.16, *p* = 0.267	*d* = −0.578	0.666	*t*(14) = −1.15, *p* = 0.272	*d* = −0.572	0.66	*t*(14) = −0.46, *p* = 0.652	*d* = −0.231	0.46
93	Hippocampus_R	*t*(14) = −1.73, *p* = 0.106	*d* = −0.865	1.107	*t*(14) = −2.17, *p* = 0.048	*d* = −1.085	1.805	*t*(14) = −2.79, *p* = 0.014	*d* = −1.394	4.013
94	Hippocampus_R	*U* = 11, *p* = 0.028	r = −0.656	5.544	*t*(10) = −3.052, *p* = 0.012	*d* = −1.526	5.8	*U* = 10, *p* = 0.021	*r* = 0.021	2.288
95	ParaHippocampal_R	*t*(14) = −1.69, *p* = 0.113	*d* = −0.847	1.067	*t*(14) = −1.71, *p* = 0.109	*d* = −0.855	1.085	*t*(9.44) = −1.946, *p* = 0.082	*d* = −0.973	1.395
96	ParaHippocampal_R	*t*(14) = −1.7, *p* = 0.112	*d* = −0.847	1.068	*t*(14) = −2.25, *p* = 0.041	*d* = −1.124	1.987	*t*(14) = −2.2, *p* = 0.045	*d* = −1.102	1.883
97	ParaHippocampal_R	*t*(14) = −2.11, p = 0.053	*d* = −1.055	1.682	*t*(14) = −2.24, *p* = 0.042	*d* = −1.119	1.963	*t*(9.53) = −2.18, *p* = 0.056	*d* = −1.09	1.829
99	Hippocampus_R	*t*(14) = −1.74, *p* = 0.104	*d* = −0.871	1.12	*U* = 18, *p* = 0.161	*r* = −0.438	1.423	*t*(8.04) = −2.98, *p* = 0.017	*d* = −1.49	5.237
124	Putamen_R	*t*(14) = −1.28, *p* = 0.222	*d* = −0.639	0.731	*t*(14) = −1.43, *p* = 0.174	*d* = −0.716	0.833	*U* = 36, *p* = 0.721	*r* = 0.721	0.491
126	Hippocampus_R	*t*(14) = −0.92, *p* = 0.372	*d* = −0.461	0.568	*t*(14) = −0.35, *p* = 0.735	*d* = −0.173	0.445	*t*(14) = 0.52, *p* = 0.611	*d* = 0.26	0.469
127	Hippocampus_R	*t*(14) = −2.44, *p* = 0.028	*d* = −1.221	2.535	*t*(14) = −2.15, *p* = 0.049	*d* = −1.075	1.767	*t*(14) = −1.15, *p* = 0.269	*d* = −0.576	0.663
134	Cingulum_Ant_L	*t*(11.41) = −1.598, *p* = 0.137	*d* = −0.799	0.971	*t*(14) = −2.1, *p* = 0.055	*d* = −1.049	1.66	*t*(14) = −1.05, *p* = 0.311	*d* = −0.526	0.618
138	Frontal_Mid_Orb_L	*t*(14) = −0.78, *p* = 0.447	*d* = −0.391	0.526	*t*(14) = −0.884, *p* = 0.398	*d* = −0.442	0.556	*t*(14) = 0.51, *p* = 0.622	*d* = 0.252	0.466
140	Frontal_Sup_Medial_L	*t*(14) = −0.85, *p* = 0.412	*d* = −0.422	0.544	*t*(14) = −1.288, *p* = 0.227	*d* = −0.644	0.737	*U* = 34, *p* = 0.878	*r* = 0.878	0.433
153	Frontal_Inf_Orb_L Insula_L	*t*(14) = −1.04, *p* = 0.315	*d* = −0.521	0.614	*t*(14) = −1.61, *p* = 0.13	*d* = −0.805	0.982	*t*(14) = −1.15, *p* = 0.268	*d* = −0.577	0.664
155	Frontal_Inf_Tri_L	*t*(14) = −1.38, *p* = 0.189	*d* = −0.691	0.797	*t*(14) = −1.35, *p* = 0.197	*d* = −0.677	0.778	*t*(14) = −0.91, *p* = 0.379	*d* = −0.454	0.564
168	Rolandic_Oper_L	*t*(14) = −0.53, *p* = 0.608	*d* = −0.263	0.47	*t*(14) = −0.05, *p* = 0.958	*d* = −0.027	0.428	*t*(14) = 1.76 *p* = 0.101	*d* = 0.879	1.139
169	Insula_L	*t*(14) = −1.33, *p* = 0.203	*d* = −0.667	0.765	*t*(14) = −1.85, *p* = 0.086	*d* = −0.924	1.253	*U* = 8, *p* = 0.01	*r* = 0.01	2.02
170	Insula_L	*t*(14) = −0.82, *p* = 0.428	*d* = −0.408	0.535	*t*(14) = −0.73, *p* = 0.478	*d* = −0.365	0.512	*t*(14) = −0.18, *p* = 0.861	*d* = −0.089	0.432
173	Insula_L	*t*(14) = −0.81, *p* = 0.43	*d* = −0.407	0.534	*t*(14) = −1.13, *p* = 0.279	*d* = −0.563	0.651	*t*(14) = −0.61, *p* = 0.549	*d* = −0.307	0.486
219	Cingulum_Ant_L	*t*(14) = −1.16, *p* = 0.266	*d* = −0.579	0.666	*U* = 21, *p* = 0.279	*r* = −0.344	0.689	*U* = 36, *p* = 0.721	*r* = 0.721	0.436
228	Amygdala_L	*t*(14) = −0.85, *p* = 0.407	*d* = −0.427	0.546	*t*(14) = −0.9, *p* = 0.381	*d* = −0.452	0.562	*t*(14) = −0.92, *p* = 0.371	*d* = −0.462	0.569
229	Hippocampus_L	*t*(9.95) = −2.467, *p* = 0.033	*d* = −1.233	2.613	*t*(14) = −2.68, *p* = 0.018	*d* = −1.341	3.47	*t*(14) = −1.58, *p* = 0.137	*d* = −0.79	0.953
230	Hippocampus_L	*U* = 3, *p* = 0.001	r = −0.906	10.483	*t*(9.53) = −4.545, *p* = 0.001	*d* = −2.273	54.484	*t*(14) = −4.2, *p* = <0.001	*d* = −2.098	31.897
231	Hippocampus_L	*t*(14) = −1.56, *p* = 0.141	*d* = −0.781	0.938	*t*(14) = −1.76, *p* = 0.101	*d* = −0.878	1.136	*t*(14) = −1.32, *p* = 0.207	*d* = −0.662	0.759
232	Hippocampus_L	*t*(9.91) = −2.129, *p* = 0.059	*d* = −1.064	1.721	*t*(14) = −2.4, *p* = .0.031	*d* = −1.201	2.407	*t*(7.91) = −2.982, *p* = 0.018	*d* = −1.491	5.252
233	ParaHippocampal_L Hippocampus_L	*U* = 10, *p* = 0.021	r = −0.688	3.948	*t*(14) = −2.155, *p* = 0.053	*d* = −1.078	1.776	*t*(9.34) = −2.311, *p* = 0.045	*d* = −1.155	2.146
235	ParaHippocampal_L	*t*(14) = −1.36, *p* = 0.195	*d* = −0.68	0.782	*t*(14) = −1.53, *p* = 0.15	*d* = −0.762	0.906	*U* = 20, *p* = 0.234	*r* = 0.234	0.836
259	Caudate_L Putamen_L	*U* = 9, *p* = 0.015	r = −0.719	4.806	*t*(14) = −2.74, *p* = 0.016	*d* = −1.367	3.728	*t*(14) = −1.84, *p* = 0.088	*d* = −0.918	1.237
262	Hippocampus_L	*t*(14) = −0.34, *p* = 0.736	*d* = −0.172	0.445	*t*(14) = −0.04, *p* = 0.967	*d* = −0.021	0.428	*t*(14) = 0.46, *p* = 0.655	*d* = 0.228	0.459
263	Thalamus_L	*t*(14) = −1.58, *p* = 0.136	*d* = −0.79	0.954	*t*(14) = −1.47, *p* = 0.163	*d* = −0.737	0.864	*t*(9.08) = −0.951, *p* = 0.366	*d* = −0.475	0.578

^a^Results correspond to the (1) Student’s *t*-test when both the assumptions of a normal distribution and equality of variances were met, (2) Welch’s statistic when only the assumption of a normal distribution was met, and (3) Mann-Whitney *U*-test when the assumption of a normal distribution was not met. ^b^Effect size is reported as Cohen’s *d* (if a parametric test was performed) or *r* (if a nonparametric test was performed); BF_10_: Bayes factor representing the likelihood of the alternative hypothesis over the null hypothesis. Correction for multiple comparisons was not performed in the frequentist analysis.

### Analysis of the results from the cold stressor challenge

**Figure 2:**
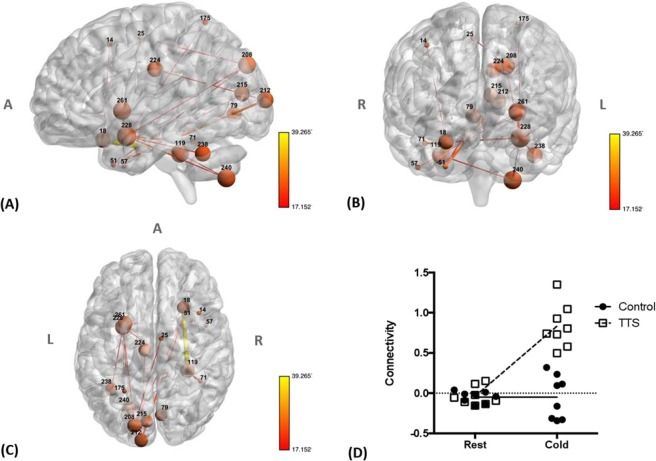
(**A**) Sagittal, (**B**) coronal and (**C**) axial views of the network identified using NBS with a mixed-design ANOVA (edge *p/F*-threshold = 0.001/17.14, network *p* = 0.027 and edge *p/t*-threshold = 0.0005/20.24, network *p* = 0.049) show increased connectivity in patients with TTS as compared to controls during the cold pressor challenge. Lines (edges) represent functional connections. Brown spheres represent the centroid of each node and are scaled according to the sum of t-statistic values over all of its significant connections. Hotter colors for connections indicate increasing statistical significance. A – anterior, R – right, L – left. (For further details, see Supplemental Table 2). (**D**) Plots showing functional connectivity (the average z-transformed correlation coefficients for the significant networks) in patients with TTS and controls during the pre-task baseline and cold pressor challenge period, respectively. Error bars indicate standard errors.

To test the hypothesis that TTS patients might have a different response to stress, a mixed-design ANOVA was used to compare functional connectivity during pre-task baseline and cold stimulus periods (within subject factor) as well as between control and TTS groups (between subject factor). This comparison revealed a network with increased functional connectivity in patients with TTS during the cold stressor challenge compared to both patients with TTS at rest and control subjects in response to cold exposure at the two higher sensitivity thresholds (edge *p/F*-threshold = 0.001/17.14, network *p* = 0.027 and edge *p/t*-threshold = 0.0005/20.24, network *p* = 0.049) (Fig. 2, Table 5, and Supplemental Material 2). The network was primarily composed of the following nodes: right insular and frontal inferior cortices (#18), right medial temporal cortex (#51), left superior occipital cortex (#208 and #212), left amygdala (#228), right (#119) and left (#238 and #240) cerebellum and left putamen (#261). Among the aforementioned nodes, the left amygdala (#228) showed the highest number of connections affected by TTS, suggesting a central role for this node in the defined network. Its connections reflected increased functional connectivity with the left putamen (#261), right inferior temporal cortex (#51), left cerebellum (#238 and #240) and left parietal (#175) and occipital (#208) lobes (Table 6). Regarding the graph theory metrics during the cold exposure task, the Bayesian analysis provided moderate or near-to-moderate evidence for the alternative hypothesis for clustering (*BF*_10_ = 3.34) and the degree (*BF*_10_ = 5.86) in ROI #263 and for the degree in ROI #140 (*BF*_10_ = 6.31).

**Table 5:** NBS results for the cold task exposure using a mixed-design ANOVA.

*Edge p/F*-threshold (df = 1,14)	Network *p*	*n* edges	*n* nodes	*η* ^2^
0.005/11.06	0.057	160	127	0.39
0.001/17.14	0.027	16	17	0.35
0.0005/20.24	0.049	6	7	0.30

df = degrees of freedom, *n* edges = number of edges in the network, *n* nodes = number of nodes in the network.

**Table 6:** Most relevant nodes and connections of the NBS resting state and cold challenge networks.

ROIs	AAL Label	MNI coordinates of the centroid voxel (mm)	Connections
**Resting state network**			
#54	Temporal_Sup_R	(50, −34, −1)	#49, #61, #85, #113, #128, #146, #169, #183, #219, #229, #259
#169	Insula_L	(−39, 8, −5)	#232, #54, #113
#219	Frontal_Sup_Medial_L Cingulum_Ant_L	(−6, 34, 26)	#54
#229	Hippocampus_L	(−22, −36, 6)	#54, #96, #183, #193, #235
**Cold challenge network**			
#18	Frontal_Inf_Orb_R Insula_R	(26, 20, −21)	#14, #119
#119	Cerebelum_4_5_R Cerebelum_6_R	(30, −36, −31)	#18, #240
#228	Amygdala_L	(−27, 2, −19)	#51, 175, #208, #238, #240, #261

ROIs = regions of interest, R = right, L = left, AAL = Automated Anatomic Label, MNI = Montreal Neurological Institute.

### Volumetric analysis

In order to assess the possibility of structural differences in our cohort, morphometric measures of volume, surface area and cortical thickness were calculated for each subject using Freesurfer. Comparisons between the TTS and control groups did not reveal significant differences (after correction for multiple comparisons or trends).

## Discussion

In this study, we analyzed the brain functional connectivity in a group of patients who had previously experienced an episode of TTS both during rest and during stress (cold exposure). In the resting state, we observed increased functional connectivity in a network composed of several regions known to be involved in emotional and autonomic control. The network components were the left anterior insula, left aCC, superior temporal cortices, left inferior frontal cortex, left hippocampus and left parahippocampal cortex. The findings are consistent with other studies showing functional alterations in patients with TTS^13^. Our team has previously shown that certain areas of the central autonomic nervous system display an altered blood-oxygen-level dependent (BOLD) response during the Valsalva maneuver^12^. While the findings from that study generally supported the hypothesis that the functional organization of the central ANS differs between controls and patients who have experienced an episode of TTS, it had two major limitations. First, only a task analysis was performed and, second, only regions known to be part of the central ANS were studied. We conducted this exploratory study to address these limitations and to expand the scope of our investigation; we analyzed functional connectivity at the whole brain level during both the resting state and a cold exposure stressor task.

Although almost all of the regions identified in the network have been previously shown to be involved in emotional and/or autonomic regulation, the insular cortex is of particular interest. It is functionally connected with the aCC, amygdala, hypothalamus and brain stem and is considered the area that is primarily responsible for integrating emotional, cognitive and social stimuli in the autonomic response^16^. Furthermore, insular cortex strokes have been shown to be associated with arrhythmias^17,18^, elevated troponin levels^19^ and other adverse cardiac events^20,21^. These studies are accompanied by a growing number of reports of the induction of TTS following ischemic stroke in both the right^9^ and left insular cortex^9–11^. One interpretation of these data is that the intrinsic activity of the insular cortex as well as its functional connectivity with other ANS areas, may play significant roles in the etiology of TTS through the overstimulation of the sympathetic network system.

The prominence of the right superior temporal gyrus in this network is also worth noting. The temporal lobe participates in processing emotional stimuli by storing previous sensory experiences^22^. In depressed patients, increased activity of the superior temporal gyrus is associated with self-referential thoughts^23^, rumination^24^, greater responses to a negative stimulus^25^ and suicidal tendencies^26^. In this context, increased functional connectivity between this area and the central ANS in patients with TTS might contribute to abnormal autonomic responses when subjects are recalling past negative experiences or experiencing modest psychological stressors.

The results of our study also suggest a potential role for the salience network, which is primarily composed of the anterior insula, dorsal aCC, amygdala, ventral striatum, dorsomedial thalamus, hypothalamus and temporal pole. This network, which was first described in 2007^27^, is responsible for self-awareness and the rapid integration of sensory or emotional stimuli to redirect attention and change behavior^28,29^. Importantly, disruption of this network has also been reported to impact on the functions of other resting state networks^29^.

Another interesting finding of our study is the predominance of left-sided structures in the network with increased functional connectivity observed in patients with TTS. This left-sided predominance would be consistent with a possible lateralization of the central autonomic nervous system that has already been described in other studies (for a review, see the study by Palma *et al*.^30^). However, some contradictory fMRI findings have been reported. In some studies, the left hemisphere was postulated to be responsible for the sympathetic system, whereas other studies suggested the opposite result^30^. The lateralization of the autonomic control is still unresolved, but future studies addressing this topic may help provide insights facilitating the interpretation of the data from the current report.

In the present study, GTA revealed increased local efficiency (capacity for information transfer), clustering coefficient (density of connections among topological neighbors) and strength (sum of the weights of the edges connected to the node) in several ROIs of the bilateral hippocampus. The hippocampus is a core component of the limbic system, which is responsible for the processing of emotional stimuli and memory formation^31^. In recent years, several authors have highlighted the role of the hippocampus in central autonomic modulation^32^, while other researchers have also suggested a role for the hippocampus in TTS^14,33^. In fact, individuals who had experienced stressful life events prior to the onset of emotional disorders (e.g., depression) display increased activity in brain regions involved in emotional perception, memory and experience^34^. For this reason, a reasonable speculation is that these subjects may be more likely to retrieve negative emotional memories, potentially rendering them more susceptible to an exacerbated central autonomic response to stress.

Given the acknowledged importance of the sympathetic-excitatory stress response in patients with TTS, we explored the central autonomic network during exposure to cold, a challenge known to increase sympathetic outflow^35^. When individuals in the TTS group were stressed, we observed increased functional connectivity in a network comprised of nodes located in the amygdala, left putamen, right insula and right cerebellum, among others, compared to patients with TTS at rest and to normal controls. The amygdala, which is responsible for the detection and integration of sensory and emotional stimuli, is a core component of the salience network and serves as a major source of inputs to the insular cortex^36^. One meta-analysis of functional brain imaging studies identified the left amygdala, right anterior and left posterior insula and mid-cingulate cortices as the core of the central autonomic network^37^. In that study, the left amygdala played a dual role, regulating both sympathetic and parasympathetic activity. Similarly, a meta-analysis of heart rate variability-related brain activity also showed a left dominance of amygdala on autonomic function^38^. On the other hand, in the present study, this network also included the right insula. Increased resting state functional connectivity in a network involving these nodes suggests the presence of abnormal autonomic modulation, including the sympathetic outflow, in response to stress. These findings further support the hypothesis that a functional signature in the central ANS exists in patients with TTS.

Volumetric analyses did not reveal differences between the TTS group and controls. Although this result is consistent with previous findings^12,13^, a recent study, based on a larger sample size, revealed significant structural differences in the limbic network in patients with TTS^15^.

Finally, we recognize a number of limitations in this study that must be considered: the small sample size (due to the rarity of the condition) and the large number of excluded patients. Nevertheless, the characteristics of our cohort were very similar to other cohorts reported in the literature, including clinical presentation and diagnostic features^39^. We chose to confine our study to female patients, because the inclusion of males in the analysis would add another confounding factor. In addition, TTS events in men are usually secondary to a severe critical illness, as was the case in our database, as opposed to the preponderance of an emotional trigger in women^39^. Patients with TTS in our cohort had a statistically significant higher incidence of depressive symptoms and tended to report more anxiety-related symptoms and perceived stress than controls. Although these differences between groups might possibly confound the fMRI results, we did not identify an association between HADS or PSS scores and NBS results. Furthermore, according to Nayeri *et al*., a pre-existing psychiatric illness is associated with increased risk of recurrent TTS^40^.

In conclusion, we observed altered central autonomic connectivity in patients with TTS both at rest and in response to a cold challenge. These findings may underlie the increased sensitivity of these subjects to acute heart failure when exposed to stressful triggers.

## Methods

### Ethics statement

This study complied with the principles specified by the Declaration of Helsinki. Approval for this study was obtained from the Ethics Committee from both the Hospital of Braga and the Life and Health Sciences Research Institute. Informed written consent was obtained prior to data collection, and subjects were allowed to withdraw from the study at any time.

### Participants and study design

This study utilized a case-control design. All patients were selected from a database of subjects admitted to the Hospital of Braga with a diagnosis of TTS between 2009 and 2015. The patients in the database had previously completed a psychological evaluation to assess depression, anxiety and perceived stress using the Hospital Anxiety and Depression Scale (HADS) and the Perceived Stress Scale – 10 item (PSS-10). Both scales have previously been validated for the Portuguese population^41,42^. Exclusion criteria were as follows: age >85 years, a high frailty index, loss to follow-up or refusal to participate in this study. The control subjects were selected from the general population of the region of Braga. Ultimately, eight patients with TTS (all female, mean age = 58.6 years, SD = 7.44) and eight sex- and age-matched controls (mean age = 58.6 years, SD = 7.50) were included and underwent an MRI evaluation. The mean interval between the TTS event and the acquisition of the MRI data was 36 months (SD = 23.63 months). Subjects were not controlled for handedness.

### MRI data acquisition

Imaging data were collected on a clinically approved Magnetom Avanto 1.5 T (Siemens, Erlangen, Germany) MRI scanner at the Hospital of Braga, using a Siemens 12-channel receiver-only head coil. First, a 3D T1-weighted magnetization prepared rapid gradient echo (MPRAGE) structural scan was acquired with the following parameters: repetition time (TR) = 2.730s, echo time (TE) = 3.48 ms, flip angle (FA) = 7°, 176 sagittal slices, in-plane resolution = 1.0 × 1.0 mm^2^ and slice thickness = 1.0 mm. This scan was followed by the acquisition of two echo planar imaging sequences sensitive to BOLD signal. For the first functional acquisition (resting state), the following parameters were used: TR = 2.0 s, TE = 30 ms, FA = 90°, resolution of 3.5 mm × 3.5 mm × 4 mm and 190 repetitions. The subjects were instructed to remain still, awake, with their eyes closed, as motionless as possible and to try to think of nothing in particular for 6 minutes. Following the scan, all participants confirmed that they had not fallen asleep. For the second functional acquisition, the following parameters were used: TR = 2.5 s, TE = 30 ms, FA = 90°, isometric resolution of 3 mm and 96 repetitions. During this acquisition, ice blocks were placed against the subjects’ left leg for a 60 s period (as a cold challenge). The task was preceded by a 120 s baseline period and followed by an additional 60s baseline period.

### MRI data preprocessing

A neuroradiologist visually inspected all images to confirm that they had not been affected by critical head motion and that participants had no brain lesions or other structural pathology. Preprocessing of functional datasets was performed using FSL (https://www.fmirb.ox.ac.uk/fsl) and included: (i) removal of the first five volumes; (ii) slice timing correction using the *slicetimer* command and interleaved slice order; (iii) motion correction using the *mcflirt* command, with the average volume as reference and the default options and motion outliers detection; (iv) skull stripping of the mean image of the functional and of the structural acquisition using *bet*; (v) linear regression of motion parameters, motion outliers and average white matter and cerebrospinal fluid signals; (vi) nonlinear registration of the structural scan to the Montreal Neurological Institute (MNI) T1 template using the *fnirt* command, in which an affine registration matrix between the two images calculated using *flirt* was used to approximate the initial registration; (vii) linear coregistration between the mean functional image and the structural image using *flirt* with 6 degrees of freedom; (viii) nonlinear transformation of the functional acquisition to MNI standard space through the sequential application of a rigid-body transformation from the functional to the structural space and the nonlinear warp calculated in the previous steps followed by resampling to a 2 mm isotropic voxel size; and (ix) bandpass temporal filtering (0.01–0.08 Hz) of the data using *fslmaths*. Excessive motion was not detected in any of the individuals studied^43^.

### MRI data analysis

Resting state data (first functional acquisition) were analyzed using a connectomics approach. Networks of connectivity were built using a functional atlas composed of 268 ROIs^44^. The mean signal over time was extracted for each ROI from each subject, and global connectivity networks for each subject were computed by calculating the Pearson correlation coefficient, followed by the Fisher’s z-transformation to assure a normal distribution.

Resting state data were analyzed using the NBS approach^45^. While NBS allows a global exploration of changes throughout the brain, important affected areas that are isolated from the network may be excluded from the results. The NBS approach was complemented with a graph theory analysis (GTA) to evaluate the network metrics and obtain a better understanding of the dynamic organization of the network.

The graph metrics properties of a subset of 41 ROIs, which included ROIs matching regions reported to be of significance in earlier reviews^30^ (the insula, aCC, amygdala, hippocampus, para-hippocampal cortex, temporal cortex, thalamus and putamen), were determined. These ROIs were extracted from the Shen atlas. The local clustering coefficient, local efficiency and nodal strength were calculated for each ROI from each subject using the Brain Connectivity Toolbox (https://sites.google.com/site/bctnet/)^46^. The local clustering coefficient is a segregation measure that is determined by the number of connections between the nearest neighbors of a node (as well as nodes that are also neighbors of one another) as a proportion of the maximum number of possible connections. The path length is defined by the minimum number of edges that must be navigated to go from one node to another. Accordingly, the local efficiency is another segregation measure and is the average inverse shortest path length in the neighborhood of the node. Finally, the nodal strength is a measure of centrality, which reflects the tendency of a node to interact with others and is calculated as the sum of the strength of all of its connections^46,47^.

A similar strategy was used to analyze the data from the cold challenge task (second functional acquisition), with the exception that the data were divided and used to build two different networks of connectivity. One corresponded to the first 120s (the pre-task baseline period) and the other to the 60s of cold exposure, thus allowing a comparison of functional connectivity between the two conditions. As described for the resting state data, the analytical pipeline was implemented with a combination of the NBS approach and GTA.

Potential structural differences between patients with TTS and control subjects were evaluated using a surface-based morphometric technique [Freesurfer (https://surfer.nmr.mgh.harvard.edu/)^48^]. Freesurfer has been extensively validated against manual segmentation^49^ and across multiple platforms and field strengths^31^. The cortical gray matter volumes, areas and cortical thickness from the Destrieux atlas^50^ and ROIs and subcortical areas from the automatic subcortical segmentation^49^ were used (160) for this analysis, and intracranial volume was used as a confounder. The processing pipeline followed the standards recommended by Freesurfer for the reconstruction and quality control workflow (https://surfer.nmr.mgh.harvard.edu/fswiki).

### Statistical analysis

The normality assumption was verified for demographic (years of education) and clinical (HADS and PPS-10 scores) variables of interest using the Kolmogorov-Smirnov (KS) test. According to the significance of the KS statistics, between-group comparisons of metric variables were performed. The results are presented as the means ± standard deviations (or medians [interquartile ranges]) for non-normally distributed variables). Statistical significance was defined at a two-tailed level of p < 0.05.

Statistical analyses of the whole brain connectomes between subjects with TTS and healthy controls was performed using the NBS method (https://sites.google.com/site/bctnet/comparison/nbs)^45^. NBS was chosen because it facilitates the identification of patterns of altered connectivity, which can extend beyond an individual connection. It is thus able to better detect alterations in wide networks and is also capable of revealing strong local effects. Briefly, NBS testing is performed in two steps. First, the hypothesis is tested for every possible connection and thresholded at a user-defined significance. Second, the resulting subnetworks (groups of regions interconnected by significantly affected connections) and their size are calculated. Finally, 5000 random permutations of the groups are performed, to which the same statistical testing of individual connections and subnetwork size calculations are applied and the significance of the initial subnetworks is determined against its distribution and corrected for the familywise error rate. Because different thresholds of connection significance will affect the possible size of the subnetworks identified, the creators of the tool suggest that several thresholds should be explored. In the present study, the thresholds of *p* = 0.005, 0.001 and 0.0005 were used to calculate the corresponding *t* and *F* values to be input into NBS. When equivalent networks survived different thresholds, the characterization was focused on the network presenting the greatest level of significance. The subnetworks were considered significant at *p* < 0.05. Comparisons of the resting state (first functional acquisition) connectomes between the two groups were performed using two samples t-tests. The significance of differences observed in the cold stress task (second functional acquisition) was analyzed using a mixed-design ANOVA, with group (*i*.*e*., TTS vs HC) as the between-subject factor and the connectivity matrices for each period (pre-task baseline vs cold exposure task) as the within-subject factor. Between-group comparisons of graph metrics were performed with independent samples t-tests. Mixed-design ANOVA and t-tests were conducted using both frequentist (without correction for multiple comparisons) and Bayesian approaches. This strategy allowed us to identify uncorrected trends for the statistical significance (the frequentist approach), with an alternative that is less dependent on multiple comparisons (the Bayesian approach)^51^. For the former, the Shapiro-Wilk test of normality and Levene’s test of the equality of variances were implemented to statistically assess the assumptions for the independent samples t-test. Welch-corrected statistics were reported for the ROIs with nonequal between-group variances. For the ROIs not meeting the assumption of a normal distribution, the Mann-Whitney test was used to perform between-group comparisons. The alternative hypothesis was defined as follows: H1 – the groups display different scores on the graph metric *g* for the ROI *r*. Bayesian analyses were implemented, considering a zero-centered Cauchy distribution –, i.e., considering a uniform default prior distribution with a scaling factor of 0.707 – which was proposed to be a reasonable threshold in most contexts^52^. Using this strategy, any value over a given range is considered equally likely. For the mixed-design models, the evidence for the interaction was tested by comparing the models with the interaction effects against the models with only the main effects (*i*.*e*., group and condition). The ratio between the marginal likelihoods of the alternative and null models – Bayes factor (*BF*_10_) – was interpreted according to Jeffrey’s thresholds: anecdotal (*BF*_10_ between 1 and 3), moderate (*BF*_10_ between 3 and 10), strong (*BF*_10_ between 10 and 30), very strong (*BF*_10_ between 30 and 100) or extreme (*BF* > 100) relative evidence. The statistical analysis of graph metrics was performed with JASP (version 0.9).

Statistical analyses of the ROI volume, area and cortical thickness obtained from Freesurfer were performed using MATLAB and corrected for multiple comparisons using the false discovery rate (FDR); a *p-*value less than 0.05 was considered significant, and a trend was considered for an uncorrected *p*-value of less than 0.01.

## Supplementary information

Supplementary Info
