# Supplementary material for: Brain functional connectivity is altered in patients with Takotsubo Syndrome
Source: Sci Rep. 2019 Mar 12;9:4187. doi: 10.1038/s41598-019-40695-3 (PMC6414524; doi:10.1038/s41598-019-40695-3)
Supplement: Supplementary file 1 — Supplementary Info [file 41598_2019_40695_MOESM1_ESM.pdf]

Brain functional connectivity is altered in patients with Takotsubo Syndrome

Ana Rita Silva[1,2], M.D., Ricardo Magalhães[1,2,5], Ph.D., Carina Arantes[3], M.D., Pedro Moreira[1,2,5], Ph.D., Mariana Rodrigues[1,2,4], Ph.D., Paulo Marques[1,2,5], Ph.D., Jorge Marques[3], M.D., Nuno Sousa[1,2,5], M.D., Ph.D., Vitor Hugo Pereira[1,2,5*,] M.D., Ph.D.

[1]Life and Health Sciences Research Institute (ICVS), School of Medicine, University of Minho, Braga, Portugal

[2]ICVS/3B's - PT Government Associate Laboratory, Braga, Portugal

[3]Cardiology Department, Hospital of Braga, Braga, Portugal

[4]Algoritmi Centre, University of Minho, Braga, Portugal

5Clinical Academic Center (2CA – Braga), Braga, Portugal]

***Corresponding Author:**

Prof. Vitor Hugo Pereira

Instituto de Ciências da Vida e Saúde (ICVS), Campus de Gualtar, Universidade do Minho, 4710-057 Braga, Portugal

+351 253 604806

+351 253 604809

vitorpereira@med.uminho.pt

Supplemental Material

Supplemental Table 1 | List of ROIs, AAL label and respective number of edges revealed by NBS analysis during rs-fMRI acquisition.

ROIs	AAL Label	MNI Coordinates	Number of Edges
#49	Occipital_Mid_R<br>Angular_R	(41,-75,-27)	1
#54	Temporal_Sup_R	(50,-34,-1)	11
#61	Rolandic_Oper_R<br>Heschl_R<br>Temporal_Sup_R	(59,-3,3)	2
#85	Cingulum_Mid_R<br>Cingulum_Post_R	(5,-39,27)	1
#96	ParaHippocampal_R<br>Fusiform_R	(29,-19,-26)	1
#113	Cerebelum_Crus1_R	(37,-57,-33)	2
#128	Caudate_R	(6,-10,5)	3
#146	Frontal_Sup_L	(-27,34,36)	1
#151	Frontal_Inf_Tri_L<br>Frontal_Inf_Orb_L	(-46,28,-7)	3
#155	Frontal_Inf_Tri_L	(-32,22,6)	1
#169	Insula_L	(-39,8,-5)	3
#183	SupraMarginal_L<br>Angular_L<br>Temporal_Sup_L	(-51,-56,20)	2
#191	Temporal_Sup_L	(-59,-30,4)	1
#193	Temporal_Mid_L	(-60,-27,-18)	1
#203	Occipital_Mid_L	(-41,-75,23)	1

#219	Frontal_Sup_Medial_L<br>Cingulum_Ant_L	(-6,34,26)	1
#229	Hippocampus_L	(-21,-37,6)	5
#232	Hippocampus_L<br>Fusiform_L	(-36,-25,15)	3
#235	ParaHippocampal_L	(-22,-4,-29)	1
#258	Caudate_L	(-12,12,8)	1
#259	Olfactory_L<br>Caudate_L<br>Putamen_L	(-10,11,-8)	1

Supplemental Table 2 | List of ROIs, AAL label and respective number of edges revealed by NBS analysis during cold exposure

ROIs	AAL Label	MNI Coordinates	Number of Edges
#14	Precentral_R<br>Frontal_Mid_R	(46,15,48)	1
#18	Frontal_Inf_Orb_R<br>Insula_R	(27,20,-21)	2
#25	Supp_Motor_Area_R	(7,-8,53)	1
#51	Fusiform_R<br>Temporal_Pole_Mid_R	(27,12,-39)	4
#57	Temporal_Pole_Mid_R	(47,4,-40)	1
#71	Fusiform_R<br>Temporal_Inf_R	(42,-46,-23)	1
#79	Lingual_R	(7,-76,-3)	1
#119	Cerebelum_4_5_R<br>Cerebelum_6_R	(30,36,-31)	2
#175	Postcentral_L<br>Parietal_Sup_L<br>Parietal_Inf_L	(-25,-55,64)	1
#208	Cuneus_L<br>Occipital_Sup_L<br>Occipital_Mid_L	(-16,-85,33)	3
#212	Calcarine_L<br>Cuneus_L<br>Occipital_Sup_L	(-11,-98,8)	2
#215	Calcarine_L<br>Cuneus_L	(-6,-81,12)	1
#224	Cingulum_Mid_L	(-7,-18,30)	1

#228	Amygdala_L	(-27,2,-19)	6
#238	Cerebelum_Crus1_L	(-37,-53,-31)	1
#240	Cerebelum_7b_L	(-21,-70,-49)	2
#261	Putamen_L	(-25,-5,0)	2
